# Exploring the roles of players in strategic purchasing for healthcare in Africa—a scoping review

**DOI:** 10.1093/heapol/czac093

**Published:** 2022-11-01

**Authors:** Mwayi Kachapila, Jesse Kigozi, Raymond Oppong

**Affiliations:** Health Economics Unit, Institute of Applied Health Research, College of Medical and Dental Sciences, University of Birmingham, Edgbaston, Birmingham B15 2TT, UK; NIHR Global Health Research Unit on Global Surgery, Institute of Translational Medicine, University of Birmingham, Mindelsohn Way Edgbaston, Birmingham B15 2TH, UK; Health Economics Unit, Institute of Applied Health Research, College of Medical and Dental Sciences, University of Birmingham, Edgbaston, Birmingham B15 2TT, UK; Health Economics Unit, Institute of Applied Health Research, College of Medical and Dental Sciences, University of Birmingham, Edgbaston, Birmingham B15 2TT, UK

**Keywords:** Strategic purchasing, Africa, scoping review

## Abstract

Following the World Health Organization (WHO) guidance on strategic purchasing in 2000, low- and middle-income countries (LMICs) are trying to shift from passive purchasing (using fixed budgets) to strategic purchasing of healthcare which ties reimbursement to outcomes. However, there is limited evidence on strategic purchasing in Africa. We conducted a scoping literature review aimed at summarizing the roles played by governments, purchasers and providers in relation to citizens/population in strategic purchasing in Africa. The review searched for scientific journal articles that contained data on strategic purchasing collected from Africa. The literature search identified 957 articles of which 80 matched the inclusion criteria and were included in the review. The study revealed that in some countries strategic purchasing has been used as a tool for healthcare reforms or for strengthening systems that were not functional under fixed budgets. However, there was some evidence of a lack of government commitment in taking leading roles and funding strategic purchasing. Further, in some countries the laws need to be revised to accommodate new arrangements that were not part of fixed budgets. The review also established that there were some obstacles within the public health systems that deterred purchasers from promoting efficiency among providers and that prevented providers from having full autonomy in decision making. As African countries strive to shift from passive to strategic purchasing of healthcare, there is need for full government commitment on strategic purchasing. There is need to further revise appropriate legal frameworks to support strategic purchasing, conduct assessments of the healthcare systems before designing strategic purchasing schemes and to sensitize the providers and citizens on their roles and entitlements respectively.

**Key messages**

- Strategic purchasing can drive healthcare reforms and strengthen systems that are dormant under fixed budgets. However, there were challenges of incorporating strategic purchasing within the public health systems. The study recommends assessment of systems before designing strategic purchasing schemes.

## Background

Purchasing is a function of healthcare financing that involves channelling funds collected from the population to healthcare providers through third parties who act as agents for the population using the pooled resources (Kutzin, 2001; Mossialos *et al.*, 2002; Figueras *et al.*, 2005). There are two types of purchasing in healthcare: first, passive purchasing, which involves funding providers using fixed budgets, and second, strategic purchasing, which seeks to maximize benefits of the pooled funds by systematically deciding what interventions to purchase (based on population needs), the best method to pay for the interventions and contracting the best providers (World Health Organization, 2010; 2013; Musgrove, 2011; Abedi *et al.*, 2018; Sanderson *et al.*, 2019). However, with respect to health financing, purchasing has been neglected compared to resource mobilization and pooling despite all the functions being equally important (World Health Organization, 2000; Hanson *et al.*, 2019).

In low- and middle-income countries (LMICs) strategic purchasing is important because it complements the efforts to attain Universal Health Coverage (UHC) by utilizing the pooled resources more efficiently (World Health Organization, 2017; Paul *et al.*, 2020; Vilcu *et al.*, 2020). However, evidence suggests that purchasing in LMICs is mainly passive and there are many challenges associated with strategic purchasing, such as fragmentation of schemes and low quality of services (Etiaba *et al.*, 2018).

Previous reviews found evidence on the implementation of strategic purchasing for healthcare in Europe, North America and Asia with little evidence of strategic purchasing for healthcare in Africa (Bastani *et al.*, 2016; Ghoddoosi Nejad *et al.*, 2017; Abedi *et al.*, 2018; Feldhaus and Mathauer, 2018). The objective of this study was to synthesize the literature on strategic purchasing in Africa by summarizing the key roles of the institutions involved. We focus on Africa because evidence shows that it has the lowest per capita spending on healthcare despite having the highest burden of disease (Sambo *et al.*, 2011). As such, strategic purchasing can be of great importance to African countries to efficiently utilize the available limited resources.

## Methods

A scoping review was conducted to identify studies with information on strategic purchasing for healthcare in Africa. Then, a descriptive analysis of the results was conducted.

### Inclusion criteria

To be included in this review, studies had to report data on strategic purchasing for healthcare in Africa. We collected data on all dimensions of strategic purchasing programmes including strategic purchasing reforms. These include purchaser–provider contracting, capitation, provider payment reforms and use of incentives such as result-based financing (RBF), alternatively called performance-based financing (PBF) or payment-for-performance (P4P). To be more inclusive, we did not limit the timeframe of interest to any period.

### Exclusion criteria

Conference abstracts, study protocols, duplicate publications, commentaries, letters, articles published in languages other than English or articles that reported on strategic purchasing for healthcare outside Africa were excluded. To collect data from quality studies, this review was limited to quantitative and qualitative studies from scientific journals.

### Search strategy

In the initial stage, we searched Embase, Ovid MEDLINE(R) In-Process & Other Non-Indexed Citations, EconLit and the World Bank Knowledge Repository electronic databases from their inception to September 2021. The search for relevant articles was based on the following keywords: ‘strategic purchasing’, ‘capitation’, ‘contracting’, ‘fee-for-service’ (FFS), ‘pay for performance’, ‘result-based financing’, ‘provider payment reforms’, ‘value-based payment’, ‘payment per case’, ‘provider contracting’, Africa, and a list of all 55 countries in Africa. FFS or payment per case were included to capture studies that might have information on schemes introduced to increase the quantity of health services. The detailed search strategies are presented in Supplementary Appendix 1. Then, we screened reference lists of additional literature reviews that were found on the topic.

### Selection of studies for the review

The screening and selection of studies adopted a multi-stage screening procedure (Roberts *et al.*, 2002). In Stage I, two reviewers independently grouped the articles into five categories based on titles, subject headings and abstracts as follows.

(a) The study reports on strategic purchasing for healthcare in Africa.
(b) The study reports on strategic purchasing for healthcare across continents including Africa.
(c) The study may have important information on strategic purchasing for healthcare in Africa but does not belong to categories (a) or (b).
(d) The study was not relevant to strategic purchasing for healthcare in Africa.
(e) The report of the study was not written in the English language.

Studies in categories (a), (b) and (c) were deemed relevant for this review and were moved forward to Stage II for further screening, while studies in categories (d) and (e) were considered not relevant for the review and were not moved forward.

### Stage II

The studies moved to Stage II were further classified into five categories after accessing and screening full papers. The two reviewers were blinded to each other and at the end disagreements were resolved by discussions. The following were categories under Stage II.

(a) Full scientific journal article on strategic purchasing for healthcare in Africa.
(b) Multi-country scientific journal article on strategic purchasing for healthcare that included data from an African country.
(c) Multi-country study that did not have data on strategic purchasing for healthcare from any African country.
(d) Conference abstract, background article or commentary.
(e) Not relevant to strategic purchasing for healthcare in Africa.

Studies in categories (a) and (b) were considered relevant and were included in the review while the rest were irrelevant and were excluded from the review. The details of the studies in each category are presented in Supplementary Appendix 2.

### Data extraction

The following study characteristics were extracted from the selected studies: names of authors, country and year of the study, analytical methods and sources of data. Then, we collected data on the key roles of the main strategic purchasing stakeholders, i.e. governments, purchasers and providers, in relation to the population/citizens.

### Data analysis

**Figure 1.**
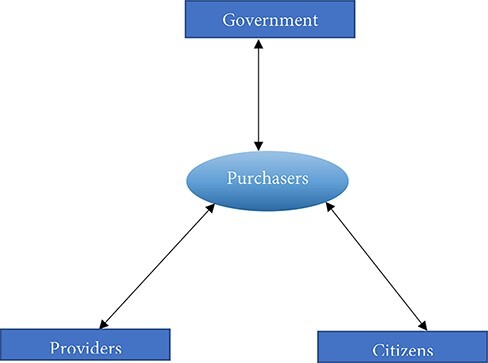
Strategic purchasing relationships between the purchasers and governments, providers and citizens

Adapted from RESYST (Resyst, 2014).

This study used an analytical framework developed by Resilient and Responsive Health Systems (RESYST). According to RESYST strategic purchasing involves relationships between the purchasers, governments and providers in relation to population/citizens where the purchasers are at the centre of the relationships (See Figure 1) (Resyst, 2014; Mbau *et al.*, 2018). RESYST identified 23 key roles of players in strategic purchasing and grouped them into three categories: key roles of government in relation to purchasers, key roles of purchasers in relation to providers and key roles of purchasers in relation to citizens or the population. This study adapted the method used by Mbau *et al.* (2018) who assessed the extent to which healthcare purchasing is strategic in Kenya by comparing the actual purchasing practices to the 23 key roles. The study summarized the published literature on strategic purchasing in Africa by focusing on the roles of players in strategic purchasing.

## Results

### Search results

**Figure 2.**
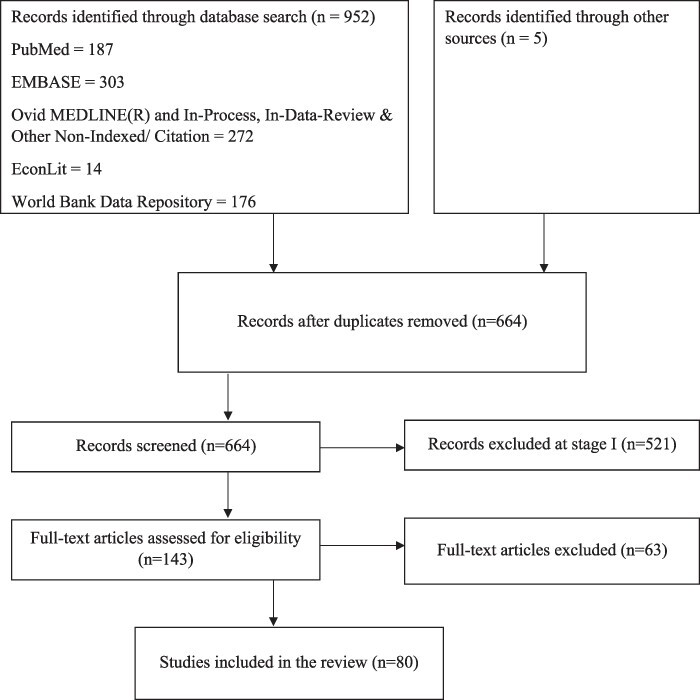
Flow diagram of the study selection process

The initial database search identified 952 potentially relevant articles. Five additional studies were identified from searching the bibliographies of 9 relevant systematic review articles identified as part of the database search (of the 952). In total there were 957 articles that were reviewed in terms of titles and abstracts of which 293 were duplicates and were excluded from the review (see Figure 2). The remaining 664 articles were then moved to Stage I of which 143 studies were moved to Stage II. At the end of the screening process 80 studies were included in the review (see Supplementary Appendix 2 for the selection process). The list of all studies included in this review are presented in Supplementary Table S1, see online Supplementary material.

### Summary of the selected studies

The studies were published between 2006 and 2021 using data from 22 of the 55 countries in Africa: Tunisia, Morocco, Egypt, Lesotho, Ghana, Burundi, Tanzania, Rwanda, Benin, Mozambique, Gambia, Burkina Faso, Uganda, Zimbabwe, Democratic Republic of Congo (DRC), Republic of Congo, Kenya, Zambia, Malawi, South Africa, Nigeria and Cameroon. This represents 40% of African countries.

### Definitions of the strategic purchasing schemes included in the review

The studies found show that in Africa strategic purchasing uses a wide range of methods namely: capitation, purchaser–provider contracting, RBF or PBF or P4P, and other schemes used a combination of the methods.

#### Capitation

Capitation is a payment method where providers are prospectively paid a flat rate for every subscriber registered on the providers’ list to cover a range of agreed services over a given period (Koduah *et al.*, 2016; Abiiro *et al.*, 2021). It is used as a cost-containment reform strategy without compromising quantity and quality of care (Aboagye, 2013; Atuoye *et al.*, 2016; Andoh-Adjei *et al.*, 2016a; 2016b; 2018a,c; Abiiro *et al.*, 2021). The evidence shows that in Ghana capitation was introduced as a cost-containing measure to FFS that was previously used to reimburse providers contracted on the National Health Insurance Scheme (NHIS) (Agyei-Baffour *et al.*, 2013; Atuoye *et al.*, 2016; Koduah *et al.*, 2016; Sackey and Amponsah, 2017; Andoh-Adjei *et al.*, 2018b,c; 2019a; Siita *et al.*, 2019). It was also used as a mechanism for ‘introducing managed competition’ between providers (Siita *et al.*, 2019, p. 2). Further, capitation was introduced to avert ‘medical shopping’ and support budgeting processes (Abiiro *et al.*, 2021, p. 873). There was also evidence of capitation in Kenya and Burkina Faso where it was used alongside FFS (Robyn *et al.*, 2014; Steenland *et al.*, 2017; Obadha *et al.*, 2020). Capitation was included in the review because by containing costs without reducing the quantity and quality of services it brings the efficiencies that are associated with strategic purchasing.

#### Purchaser–provider contracting

Purchaser–provider contracting (hereafter contracting) involves contracts between purchasers and providers that specify the services that the provider offers to the population on behalf of the purchaser (Siddiqi *et al.*, 2006). In South Africa, Lesotho, Tanzania, Tunisia, Morocco, Egypt and Mozambique contracting was used as a tool to extend services to under-served locations or to enable the public to have access to services that were offered by private-sector providers (Siddiqi *et al.*, 2006; Hongoro *et al.*, 2015; Surender *et al.*, 2016; Binyaruka *et al.*, 2018; Maluka *et al.*, 2018; Mureithi *et al.*, 2018; Rao *et al.*, 2018). Contracts that are solely based on historical budgets or contracts that are not associated with reforms are not strategic (Greer *et al.*, 2020). As such, this review included contracting schemes if the contracts were aimed at promoting efficient resource utilization or were associated with healthcare reforms. Contracts based on solely fixed budgets were excluded from the review.

#### RBF/PBF or P4P

RBF, alternatively called PBF or P4P is the payment of financial incentives to health workers or facilities after achieving agreed healthcare targets that are sometimes adjusted for quality of care (Basinga *et al.*, 2011; Honda, 2012; Witter *et al.*, 2012; Binagwaho *et al.*, 2014; Chimhutu *et al.*, 2014; 2019; Manongi *et al.*, 2014; Wilhelm *et al.*, 2016; Antony *et al.*, 2017; Chinkhumba *et al.*, 2017; 2020; Ngo *et al.*, 2017; Binyaruka *et al.*, 2018; Manga *et al.*, 2018; Fichera *et al.*, 2021; Ssengooba *et al.*, 2021). RBF was included in this review because the purchaser decides on what services to purchase, for which groups of citizens, from which providers and at what price. It is likely that RBF was the most practiced strategic purchasing method as it was reported in 50 of the 80 studies from 18 countries (see the list in Supplementary Appendix 4).

#### Mixed methods

The results show that the strategic purchasing methods mentioned above are not always used in isolation, as many countries used a combination of the methods. For example, in Nigeria FFS was combined with capitation in the NHIS that was funded by general taxation and formal sector payroll contributions (where all federal civil servants qualify for the scheme) (Mohammed *et al.*, 2014; Etiaba *et al.*, 2018). In Burkina Faso, capitation was combined with RBF while in Ghana and Kenya capitation was combined with FFS and salaries (Robyn *et al.*, 2012; Agyepong *et al.*, 2014; Obadha *et al.*, 2020).

### Key roles identified from the studies

The results are presented according to the 23 key roles of strategic purchasing played by governments, purchasers and providers in strategic purchasing, i.e. the roles played in capitation, contracting, RBF and mixed strategic purchasing methods identified above. The 23 roles (Role 1–23) are defined in Supplementary Appendix 3.

### Key roles of governments in relation to purchasers

Governments are expected to provide strategic purchasing regulatory frameworks and policy directions to facilitate the strategic purchasing arrangements (Honda and McIntyre, 2016). The findings show that with respect to establishing regulatory frameworks for purchasing for both purchasers and providers (Role 1), in some countries laws, regulations and policies were put in place by governments (Siddiqi *et al.*, 2006; Agyepong *et al.*, 2014; Vian *et al.*, 2015; Etiaba *et al.*, 2018; Maluka *et al.*, 2018; Rao *et al.*, 2018; Chansa *et al.*, 2019). For example, in Cameroon a law was passed to ensure that the purchasing agency had the purchasing rights (Sieleunou *et al.*, 2021). However, in some countries the laws needed to be further revised to accommodate new roles relating to purchasing or contracting (Siddiqi *et al.*, 2006; Sieleunou *et al.*, 2021).

Prior to implementing strategic purchasing, the Rwandan and Zambian governments undertook wide range of health-sector reforms including decentralization, which gave autonomy to local authorities and facility managers to run health facilities (Soeters *et al.*, 2006; Chansa *et al.*, 2019). In Rwanda, the roles of the fund holders, providers and national decision makers who were responsible for identifying cost-effective interventions were well defined (Soeters *et al.*, 2006). Based on the lessons from Rwanda, it was argued that RBF can be used as a tool for bringing changes to a health system that give autonomy to providers to identify services needed by the population and improve allocation of resources (Meessen *et al.*, 2006; Honda, 2012).

Governments are supposed to take a leading role in filling-in service delivery infrastructure gaps Mbau *et al.*, (2018) (Role 2). However, in many countries RBF schemes were introduced as pilot schemes with technical or financial support from development partners, especially the World Bank (de Walque *et al.*, 2013, Binagwaho *et al.*, 2014; Chimhutu *et al.*, 2014; 2016; 2019; Manongi *et al.*, 2014; Borghi *et al.*, 2015; Skiles *et al.*, 2015; Wilhelm *et al.*, 2016; Antony et al., 2017; Chinkhumba *et al.*, 2017; Sieleunou *et al.*, 2017; Binyaruka et al., 2018; Lohmann *et al.*, 2018; Mabuchi *et al.*, 2018; Manga *et al.*, 2018; Ridde *et al.*, 2018; Zeng *et al.*, 2018; De Allegri *et al.*, 2019a,b; Ssengooba *et al.*, 2021; Ssennyonjo *et al.*, 2021). In Zimbabwe donors supported the Government by pooling funds into a single fund (Witter *et al.*, 2019a).

Public schemes like national health insurance (NHI) and RBF had larger baskets of interventions compared to private purchasing arrangements like private insurance and health management organizations (HMO) (Sieleunou *et al.*, 2021). However, there were doubts about governments’ capacity or commitment to continue with strategic purchasing interventions at the end of pilot schemes (Wilhelm *et al.*, 2016; Ridde *et al.*, 2018; James *et al.*, 2020; Seppey et al., 2022).

There was evidence of governments taking leading roles in mobilizing resources for strategic purchasing (Role 3). For example, the Government of Kenya made subscription into the National Hospital Insurance Fund (NHIF) capitation scheme mandatory for all people in formal employment (Obadha *et al.*, 2020). In Burkina Faso and Nigeria, general government funding was combined with citizen payments into insurance schemes or patient payments (Mohammed *et al.*, 2014; Robyn *et al.*, 2014; Steenland *et al.*, 2017).

Governments are expected to put in place accountability processes for purchasers (Role 4). However, in some cases donors established additional accountability frameworks through their fund holders because they perceived the government accountability systems as weak (Witter *et al.*, 2019a; 2020; Sieleunou *et al.*, 2021). In Nigeria, conflicts between key stakeholders resulted in compromized purchaser–provider relationships which affected accountability and administrative structures (Ogbuabor and Onwujekwe, 2018).

### Key strategic purchasing roles of purchasers in relation to providers

Purchasers are supposed to define and regularly update a basket of cost-effective services that reflect the population’s needs, and develop and implement systematic methods for identifying and contracting providers of the services (Resyst, 2014). During service delivery, the system should provide channels to allow the providers to be accountable to the purchaser (Honda and McIntyre, 2016).

#### Selecting and contracting providers

Purchasers should select and contract providers by considering the range and quality of services and their location (Roles 5 and 6). There were formal assessments for providers against accreditation criteria and in some cases this involved competitive bidding (Vian *et al.*, 2015; Koduah *et al.*, 2016; Steenland *et al.*, 2017; Binyaruka *et al.*, 2018; Etiaba *et al.*, 2018; Witter *et al.*, 2020; Sieleunou *et al.*, 2021). For example, providers were contracted if they met minimum standards like: having a business plan, bank account, operation health facility committee (HCC), not charging user fees or providing a basket of defined services (Koduah *et al.*, 2016; Steenland *et al.*, 2017; Binyaruka *et al.*, 2018; Etiaba *et al.*, 2018; Witter *et al.*, 2020; Sieleunou *et al.*, 2021). Some schemes contracted both district health offices (who supervised the providers) and providers (Witter *et al.*, 2019a). Zambia contracted all public primary healthcare providers for 10 years in a nationwide RBF scheme (Chansa *et al.*, 2019). However, it was not clear how targets for the services were selected (Witter *et al.*, 2012). Further, once accredited the contracts were rarely reviewed for compliance (Etiaba *et al.*, 2018; Maluka *et al.*, 2018).

Some studies found that there was a lack of systematic processes for selecting providers and sometimes providers who did not meet the requirements were selected, which was attributed to lack of competition between providers in the target locations (Siddiqi *et al.*, 2006; Maluka *et al.*, 2018; Rao *et al.*, 2018; Sieleunou *et al.*, 2021). In some cases, the criteria for selecting location was not clear. For example, in Ghana, some people felt that districts that were chosen for a capitation pilot scheme were purposively selected to limit healthcare access to the population that was deemed to be opposition party supporters (Andoh-Adjei et al., 2016a; 2019b; Abiiro *et al.*, 2021).

In South Africa, the Government contracted general practitioners (GPs) as part of the NHI re-engineering process (Volmink *et al.*, 2014; Hongoro *et al.*, 2015; Mureithi *et al.*, 2018; Rao *et al.*, 2018). However, the consultations between the purchasers and the providers when designing the contracts were not adequate and as a result most GPs did not sign the contracts, whereas some contracted GPs were not well conversant with the contracts they had signed (Hongoro *et al.*, 2015). Some schemes were designed without proper knowledge of the healthcare system, resulting in inadequate finances or human resources to support schemes (Manongi *et al.*, 2014; Witter *et al.*, 2019a).

#### Managing service-delivery standards

Purchasers are expected to develop a list of medicines that providers can prescribe, medical supplies for which they will be reimbursed, and standard treatment guidelines to ensure quality of services (Role 7). Five studies established that the introduction of strategic purchasing schemes like RBF encouraged the use of standard treatment guidelines through strengthened monitoring and supervision, refresher training and verification processes (Menya *et al.*, 2015; Ogundeji *et al.*, 2016; Lohmann *et al.*, 2018; Kane *et al.*, 2019; Witter *et al.*, 2020). The Ministry of Health (MOH) acting as a purchaser developed guidelines for services in the contracts in order to standardize payments (Rao *et al.*, 2018). There were schemes that encouraged providers to improve the quality of equipment and infrastructure (Das *et al.*, 2016; Chinkhumba *et al.*, 2017). However, the authors established that the facility managers were not motivated to improve the structures because of a lack of funds (Das *et al.*, 2016).

#### Developing and managing payment methods

Purchasers are expected to establish provider payment methods and rates, ensure regular payments and monitor user payment policies to stimulate efficiency in service provision (Roles 8, 9, 14 and 17). Purchasers motivated providers to cost the services, and as a result the patients paid the right amount for the services received (Manongi *et al.*, 2014). Purchasers in Kenya and Ghana stated that reimbursement rates were calculated using data from the public health system (Menya *et al.*, 2015; Koduah *et al.*, 2016). However, the providers in Ghana claimed that the rate was set too low and threatened to pull out of the scheme (Aboagye, 2013; Andoh-Adjei *et al.*, 2016b). The authors suggested that the purchaser needed to conduct a formal costing exercise to establish the true capitation reimbursement rate (Andoh-Adjei *et al.*, 2016b).

Some RBF schemes specified a percentage of the funds that was to be rewarded to health workers while in other schemes this was at the discretion of facility managers (Basinga *et al.*, 2011; Witter et al., 2012; Ssennyonjo *et al.*, 2021). As such, some facility managers decided to reward all workers at the facility uniform amounts or based on the workers’ ranks, implying that the bonuses were not based on performance (Chimhutu *et al.*, 2014; 2016; Ogundeji *et al.*, 2016; Witter *et al.*, 2019a,b). Ogundeji *et al.*, (2016) found that this was due to managers’ lack of knowledge of the personal performance assessment system. In other countries, attainment of autonomy on finances was possible for private providers but was difficult for public providers who were mandated by law and other regulations to remit funds to higher offices or implement decisions made by central offices (Soeters *et al.*, 2006; Obadha *et al.*, 2020; Sieleunou *et al.*, 2021). Elsewhere, the funds were only to be used for facility operations (Menya *et al.*, 2015).

There were instances where schemes like RBF did not involve changing the provider payment systems but rather introduced rewards on top of the fixed budgets (Basinga *et al.*, 2011; Chimhutu *et al.*, 2016; Witter *et al.*, 2020; Paul *et al.*, 2021). Witter *et al.*, (2020, p.8) argued that there was ‘correlation’ between facility revenue and ‘catchment population’ and prices, such that the RBF was mainly an additional source of health facility funding and not a reward for performance. Some RBF schemes included rewards for facilities and health workers, including government district officers who supervised the facilities (Borghi *et al.*, 2015; Das *et al.*, 2016; Binyaruka *et al.*, 2018; Lohmann *et al.*, 2018; Binyaruka and Anselmi, 2020).

Many strategic purchasing schemes were associated with delayed payments, which affected providers’ planning and budgeting (Aboagye, 2013; Maluka *et al.*, 2018; Ogbuabor and Onwujekwe, 2018; Rao *et al.*, 2018; Andoh-Adjei *et al.*, 2018c; Kane *et al.*, 2019; Obadha *et al.*, 2019; Witter *et al.*, 2020; Abiiro *et al.*, 2021). In South Africa, after it was established that payments were being delayed because of bureaucracies of the public health system, the purchaser outsourced services to a payment company (Mureithi *et al.*, 2018; Rao *et al.*, 2018). In Ghana, there was lack of sensitization of the providers to the difference between capitation and other methods like FFS (Aboagye, 2013; Abiiro *et al.*, 2021).

#### Performance monitoring, auditing and enforcing adherence to contracts

Purchasers are expected to put in place mechanisms for sourcing information for monitoring provider performance and correcting non-adherence to protocols (Roles 10 and 11). In Kenya an online system for reporting health management information was put in place by the purchaser, but providers complained that service provision was affected by frequent system interruptions as they could not offer services without reporting (Obadha *et al.*, 2019). Maluka *et al.*, (2018) established that purchasers complained that the providers could not account for the user fees agreed in the cost-sharing contract. Providers were not necessarily pricing the services as stipulated in the contract and some facilities continued charging user fees while on a contract that required abolishment of user fees (Maluka *et al.*, 2018; Witter *et al.*, 2020). However, there was no evidence of the purchasers correcting the non-compliance of the contract.

Elsewhere strategic purchasing implementation was monitored using existing national indicators (Basinga *et al.*, 2011; Witter *et al.*, 2020). However, some processes (including monitoring and evaluation processes) of the strategic purchasing programmes could not fit into the public health system (Wilhelm *et al.*, 2016; Witter *et al.*, 2020; Sieleunou *et al.*, 2021) (Siddiqi *et al.*, 2006; Surender *et al.*, 2016; Etiaba *et al.*, 2018; Mureithi *et al.*, 2018; Rao *et al.*, 2018). For example, contracts were poorly administered and monitored because of lack of expertise by the public purchasers (Siddiqi *et al.*, 2006; Surender *et al.*, 2016; Etiaba *et al.*, 2018; Mureithi *et al.*, 2018). Rao *et al.*, (2018), suggested that monitoring can be improved by engaging third parties to do the monitoring on behalf of the purchaser. Other authors argued that verification of services is more effective at enforcing guidelines when combined with coaching (Bertone and Meessen, 2013).

In Zambia contracting was done at a district level, as such it was difficult for the purchaser to act on poor performance by withholding payments because that would also penalize facilities that were performing well (Chansa *et al.*, 2019). In theory the verification of performance was supposed to adjust the claims depending on quality of care, but in practice there was no evidence of verifying the quality of the services provided (Sieleunou *et al.*, 2021). Some authors recommended improving monitoring by implementing a fully-fledged monitoring system with peer review committees (Mohammed *et al.*, 2014).

Purchasers are expected to put in place mechanisms for monitoring, auditing and preventing fraud, including securing information systems (Roles 12, 13 and 18). It was reported that there was verification of claims before reimbursing the providers (Basinga *et al.*, 2011; Antony *et al.*, 2017; Rajkotia *et al.*, 2017; Sieleunou *et al.*, 2017; Binyaruka *et al.*, 2018; Binyaruka and Anselmi, 2020; Ssennyonjo *et al.*, 2021). One study found that in a demand-side RBF, verification involved checking with beneficiaries to confirm that they had received the services that were reported (Chinkhumba *et al.*, 2017). In South Africa, providers completed monthly timesheets of the services provided (Mureithi *et al.*, 2018).

In other schemes, providers were audited frequently by district officers, but clinicians and data officers complained that the audit processes utilized much of the time that could be used for consultations and data analysis respectively (Antony et al., 2017; Shen *et al.*, 2017; Witter *et al.*, 2019a). One study found that there was under-reporting of negative outcomes that were of interest to the RBF programme, suggesting that verification needed improvement (De Allegri *et al.*, 2019a). A Cochrane review established that in Zambia and Tanzania providers were doing self-assessment under the supervision of a district health management team while in Rwanda and DRC the assessments were done by external agencies (Witter *et al.*, 2012).

#### Facilitating equitable allocation of resource

Purchasers are expected to ensure that resources are allocated equitably across locations (Roles 15 and 16). Seven studies found that purchasers contracted providers who provided services to under-served communities to ensure that those communities receive services which could not be provided by the public health system (Siddiqi *et al.*, 2006; Hongoro *et al.*, 2015; Surender *et al.*, 2016; Binyaruka *et al.*, 2018; Maluka *et al.*, 2018; Mureithi *et al.*, 2018; Rao *et al.*, 2018). In Zimbabwe, the RBF scheme was designed to reimburse providers, using higher rates for providing services to the poor, in hard-to-reach areas or for reducing user fees for the poor. However, there was scanty evidence of the actual implementation of these equity components (Witter *et al.*, 2019a; 2020). RBF was implemented for maternal and child health programmes (Basinga *et al.*, 2011; Das *et al.*, 2016; Wilhelm *et al.*, 2016; Chinkhumba *et al.*, 2017; Rajkotia *et al.*, 2017; Steenland *et al.*, 2017; Wright and Eichler, 2018; Chansa *et al.*, 2019; Chinkhumba *et al.*, 2020; De Allegri *et al*., 2019a* *; James *et al*., 2020* *; Zizien *et al.*, 2019; Ferguson *et al.*, 2020). Sometimes RBF was implemented alongside policies to reduce fees for women and children who were perceived as worse-off and some schemes included equity bonuses (Ridde *et al.*, 2018; Seppey *et al.*, 2020). Some authors argued that because the schemes focused on reproductive, maternal and child health, they focused on women and children who are likely to be the less privileged, which could address the problem of unequitable distribution of healthcare (Witter *et al.*, 2019a; 2020).

### Key roles of purchasers in relation to population served or citizens

Purchasers are expected to put in place mechanisms to ensure that the services offered by the providers reflect the choices of the population being served (Honda and McIntyre, 2016). As such, purchasers are expected to assess the service needs, preferences and values of the citizens, define benefit packages, inform the population of their obligations and entitlements and ensure that the citizens access the entitlements (Roles 19–21). Further, the purchasers should establish effective complaints and feedback mechanisms and report on the use of resources and other measures of performance (Roles 22 and 23).

There was evidence of community involvement in the development and management of strategic purchasing schemes, suggesting that the needs of the population were considered. At the planning stage, communities were involved in the conceptualization, resource mobilization, designing and development of business plans, raising funds and building structures, and in some schemes it was mandatory for facilities to have a functional HCC (Soeters *et al.*, 2006; Robyn *et al.*, 2012; Manongi *et al.*, 2014; Wilhelm *et al.*, 2016; Witter *et al.*, 2019b; 2020; Ferguson *et al.*, 2020; Sieleunou *et al.*, 2021). However, it was argued that some members in the committees were compromized because they were appointed by the government. Further, the recommendations by the community could not always be implemented because the facility managers did not have decision-making autonomy (Sieleunou *et al.*, 2021).

At the implementation stage, strategic purchasing schemes enhanced processes of soliciting feedback on the services provided from the community which were dormant under fixed budgets. This was done through enhancing patient satisfaction surveys, participation in verification of claims, enforcement of health facilities actions in the community and completion of regular reports (Meessen *et al.*, 2006; Soeters *et al.*, 2006; Manongi *et al.*, 2014; Antony *et al.*, 2017; Steenland *et al.*, 2017; Fritsche and Peabody, 2018; Mabuchi *et al.*, 2018; Chimhutu *et al.*, 2019; Witter *et al.*, 2019a; 2020; Sieleunou *et al.*, 2020; Ssengooba *et al.*, 2021; Ssennyonjo *et al.*, 2021). However, there was a lack of evidence of using the feedback from the community to improve the decision making of the facilities (Witter *et al.*, 2020). Citizens’ entitlements were communicated through radio programmes (Witter *et al.*, 2020).

Witter *et al*. (2019a** pp. 10–11) found that some strategic purchasing schemes focused on the national health priorities ‘minimum healthcare package’ without considering the needs of the targeted local communities. In Ghana, some people perceived that the benefits of the NHIS were not enough, others felt that capitation ties subscribers to a single provider which limits access to emergency services, and others did not fully understand the capitation policy and received the news of policy changes through the media without being consulted (Koduah *et al.*, 2016; Andoh-Adjei *et al.*, 2018c; Abiiro *et al.*, 2021). This suggests that the citizens were not widely consulted and sensitized on the package.

In some RBF schemes there was little engagement and consultation with the community and less frequent community monitoring than stipulated in the plans, which might have limited the citizen’s access to vital information (Meessen *et al.*, 2006; Witter *et al.*, 2019b; 2020). Further, there were some disagreements between the subscribers and the providers as the providers declined to provide services to some subscribers arguing that their names were not on the capitation list (Koduah *et al.*, 2016).

RBF introduced a requirement for providers to display a list of services in a package, prices and expenditures, and introduced service charters that empowered citizens to request their entitlements. However the implementation of these accountability mechanisms was not effective because of a lack of adequate funds (Witter *et al.*, 2019b).

## Discussion

This review identified strategic purchasing roles that are played by governments, providers and purchasers in relation to citizens in Africa. The results show that to some extent purchasers and governments have used strategic purchasing to bring some health system changes and/or strengthen good practices that are dormant under fixed budgets. For example, in some countries there was evidence of laws being revised to accommodate new roles of the purchasers. However, in some countries it was mentioned that the legal frameworks needed to be updated. Purchasers used strategic purchasing to encourage soliciting society views on services offered by the facilities through strengthening heath facility committees. However, the review also identified many challenges in the relationships between the institutions, like lack of strong government stewardship, lack of coordination between purchasers, providers and governments, and implementing old systems contrary to the strategic purchasing contracts.

In many strategic purchasing schemes development partners played leading roles in mobilizing funding, depicting a lack of government stewardship at mobilizing resources for strategic purchasing. Evidence shows that in Africa there are high levels of out-of-pocket and catastrophic expenditures (Shrime *et al.*, 2015; Beogo *et al.*, 2016). As such, resource mobilization for strategic purchasing schemes is a key area that needs serious consideration to ensure that strategic purchasing schemes are scaled-up to reach out to more citizens. There are some models for resource mobilization for strategic purchasing in Africa that seem to be feasible for implementation in other countries, e.g. implementing NHIS systems to fund strategic purchasing like in Ghana, Nigeria and Kenya where governments put in place and implemented NHIS mainly funded by the citizens or civil servants.

There was a lack of evidence on incorporating feedback from the community into the management of facilities mainly because facility managers, especially public providers, do not have the authority to make decisions. As such, where possible, healthcare reforms should be implemented before rolling out strategic purchasing schemes as was done in Rwanda and Zambia (especially facility managerial autonomy reforms). Governments should make sure that there are wider consultations with all stakeholders, including purchasers and providers, to ensure that the proposed schemes are feasible given the systems in which they will be implemented. Such consultations should focus on identifying bottlenecks and reforms that need to be conducted before the schemes are implemented. Further, purchasers should identify the needs of the population and design strategic purchasing schemes that are tailored to specific locations for the strategic purchasing schemes to function properly.

Among other issues, strategic purchasing schemes were affected by delayed payments, inadequate human resources, lack of purchaser’s capacity to monitor the implementation of the schemes and time-consuming monitoring systems. A solution would be to consider revising health management information systems to automate some data processes to reduce the work-load of the providers. However, the designing of such systems needs to take into consideration the local challenges of the country or location where the strategic purchasing schemes will be implemented.

### Comparison with other studies

These findings are similar to the findings of a study that synthesized evidence mainly from Europe and North America that found that strategic purchasing was affected by lack of governments’ stewardship to create a competitive environment and lack of governments’ capacity, as the main purchasers, to manage strategic purchasing interventions (Ghoddoosi Nejad *et al.*, 2017). Our results are also consistent with findings that maternal health RBF schemes that were conducted to improve the quality of healthcare in LMICs (payment for quality of services) were associated with unreliable payments by purchasers (Wright and Eichler, 2018).

### Strengths and limitations of the study

To the best of our knowledge, this is the first review of the literature to synthesize evidence on the roles of the players in strategic purchasing for healthcare in Africa. We conducted a comprehensive review that searched a wide range of databases for quantitative and qualitative scientific journal articles on strategic purchasing and we did not limit the search to a particular period.

There are limitations to this study. First, the results from this review should be interpreted with caution because the studies were from less than half of the countries in Africa. Although the search was comprehensive, we did not find studies from more than half of the countries. Second, the findings in this study are limited to the literature extracted from the 80 studies found during the search. There may be some evidence on strategic purchasing in Africa that may not be published in scientific journal articles. Third, the results reflect the roles of governments, purchasers and providers over the past two decades, the period from which the data of the studies included in this review were collected. It should be noted that roles are not static and do evolve over time. As such, the results should be interpreted with caution, because as countries continue to implement reforms to accommodate the roles of new strategic purchasing arrangements or improve service delivery, the relationships between some of the institutions presented in the present study might change. Finally, this study aimed at summarizing how strategic purchasing is being implemented in Africa and does not include results on the impact of strategic purchasing on healthcare outcomes.

### Recommendations

Strategic purchasing involves identifying the needs of the population, selecting the most efficient healthcare providers and giving incentives to the selected providers to improve outcomes (Sanderson *et al.*, 2019). First, we recommend that before signing contracts, purchasers should design monitoring systems that enable them to monitor implementation of the contracts, and the monitoring systems should not be highly labour intensive to avoid compromising service delivery.

Second, this study recommends that prior to implementation of strategic purchasing schemes, governments and purchasers need to conduct situation analysis to identify opportunities and weaknesses to be targeted with reforms before designing strategic purchasing. This would avoid situations where the new changes proposed in the schemes cannot be implemented because of system challenges like inadequate staff or unavailability of quality data for decision making. For example, the need for more human resources or data requirements should be taken into consideration if future strategic purchasing programmes are to work. Further, purchasers need to make sure that before strategic purchasing schemes are rolled out, all providers must be sensitized on their requirements and the expectations from the new scheme. Similarly, purchasers need to put in place good communication channels to inform citizens of their entitlements and arrange for feedback mechanisms that are feasible.

Third, African countries can benefit a lot if governments are on the forefront and are committed to shift from fixed budgets to strategic purchasing, which was lacking at least based on some literature reviewed in the study. On a positive note, there were already accountability systems within the public healthcare systems which can be utilized to further improve strategic purchasing. However, there were some concerns that some government accountability frameworks were weak and these need to be strengthened.

Fourth, purchasers, governments and providers should give communities full autonomy to select members of community boards to ensure that the boards and their decisions are not influenced by the authorities that appointed them. Related to this, there is a need to decentralize decision making especially in public facilities because that would allow the facility managers to consider the decisions made by the communities through the various channels. Decentralization would also allow facility managers to have autonomy to make financial decisions, which was lacking in some strategic purchasing initiatives.

Finally, strategic purchasing is a new concept which does not fit with most policies, regulatory frameworks and public healthcare systems in many African countries. For strategic purchasing to fit in the systems there is a need to review the legislations and policies in order to identify legal framework gaps that need to be revised and new laws and policies that need to be formulated to facilitate strategic purchasing.

## Conclusion

This scoping review synthesized the literature on the roles played by governments, purchasers and providers in relation to citizens on strategic purchasing for healthcare in Africa. The findings show that strategic purchasing can be used as a tool for healthcare reforms and for strengthening systems that are dormant in fixed-budget systems. In many schemes, development partners played leading roles in terms of resources mobilization and there were challenges because some public health systems could not accommodate the proposed changes. Future designing of strategic purchasing schemes should consider conducting situation analysis before designing the schemes.

## Supplementary Material

czac093_Supp

## Reflexivity statement

This study was conducted by three authors who are at different levels of seniority and expertise. All the authors have relevant experience working in governments, and academia in African countries including Malawi, Uganda, and Ghana. They have research interests and ongoing research work in low-and middle-income countries (LMICs) including Africa. One author is an early career researcher while two have vast research experience in health economics research aimed at improving health systems in both LMICs and high-income countries. One author focuses on economic evaluations of surgical interventions in LMICs. One researcher has experience in conducting economic evaluations in the areas of musculoskeletal disease, arthritis, mental health and workplace interventions. One researcher has experience in conducting economic evaluations in the areas of antimicrobial resistance, musculoskeletal disease and multinational trials and the application of health economics to influence policy in LMICs.
