# Supplementary material for: Exploring the roles of players in strategic purchasing for healthcare in Africa—a scoping review
Source: Health Policy Plan. 2022 Nov 1;38(1):97–108. doi: 10.1093/heapol/czac093 (PMC9849715; doi:10.1093/heapol/czac093)
Supplement: czac093_Supp [file czac093_supp.zip › Supplementary files czac093.docx]

**Supplementary Appendix 1: Search mesh terms**

**Database: Embase <1974 to 2021 September 13> Search** **Strategy:**

--------------------------------------------------------------------------------

1 "Africa south of the Sahara"/ or *South Africa/ or *Africa/ or *North Africa/ or *Central Africa/ (24768)

2 (Algeria or Angola or Benin or Botswana or "Burkina Faso" or "Burkina Fasso" or "Upper Volta" or Burundi or Urundi or Cameroon or Cameroons or Cameron or Camerons or "Cape Verde" or "Central African Republic" or Chad or Comoros or "Comoro Islands" or Comores or Congo or Zaire or "Cote d'Ivoire" or "Ivory Coast").ti,ab,kw. (55008)

3 (Djibouti or Egypt or Eritrea or Ethiopia or Gabon or "Gabonese Republic" or Gambia or Ghana or "Gold Coast" or Guinea or Kenya or Lesotho or Basutoland or Liberia or Libya).ti,ab,kw. (187891)

4 (Madagascar or Malawi or Nyasaland or "Mali Marshall Islands" or Mauritania or Mauritius or "Agalega Islands" or Morocco or Mozambique or Namibia or Niger or Nigeria or "Northern Mariana Islands").ti,ab,kw. (84759)

5 (Rwanda or Ruanda or "Sao Tome" or Senegal or Seychelles or "Sierra Leone" or Somalia or Sudan or Suriname or Surinam or "South Sudan Swaziland" or Eswatini or Tanzania or Togo or "Togolese Republic" or Tunisia or Uganda or Zambia or Zimbabwe or rhodesia).ti,ab,kw. (81662)

6 1 or 2 or 3 or 4 or 5 (402375)

7 (strategic adj8 purchas*).ti,ab,kw. (149)

8 (Quality-based adj8 purchas*).ti,ab,kw. (7)

9 (Quality adj8 based adj8 purchas*).ti,ab,kw. (150)

10 (Performance-based adj8 purchas*).ti,ab,kw. (10)

11 (Performance adj8 based adj8 purchas*).ti,ab,kw. (109)

12 (result? adj8 based adj8 purchas*).ti,ab,kw. (105)

13 (result-based adj8 financ*).ti,ab,kw. (8)

14 (result? adj8 based adj8 financ*).ti,ab,kw. (341)

15 Fee-for-service.ti,ab,kw. (8043)

16 pay-for-performance.ti,ab,kw. (2822)

17 (provider-payment adj8 reform?).ti,ab,kw. (59)

18 (provider adj8 payment adj8 reform?).ti,ab,kw. (83)

19 (Strategic adj8 contract*).ti,ab,kw. (72)

20 Bundled-Paym*.ti,ab,kw. (1467)

21 (Value-based adj8 payment?).ti,ab,kw. (895)

22 (value-based adj8 purchas*).ti,ab,kw. (705)

23 (Purchas* adj8 reform?).ti,ab,kw. (134)

24 GP-contracting.ti,ab,kw. (0)

25 (GP adj8 contract*).ti,ab,kw. (286)

26 (hospital adj8 contract*).ti,ab,kw. (1657)

27 (provider adj8 contract*).ti,ab,kw. (547)

28 capitation.ti,ab,kw. (2839)

29 payment-per-case.ti,ab,kw. (21)

30 (contracting adj8 mechanism).ti,ab,kw. (99)

31 (contract* adj8 health adj8 servic*).ti,ab,kw. (775)

32 (purchas* adj8 mechanism*).ti,ab,kw. (175)

33 (contract* adj8 arrangem*).ti,ab,kw. (689)

34 7 or 8 or 9 or 10 or 11 or 12 or 13 or 14 or 15 or 16 or 17 or 18 or 19 or 20 or 21 or 22 or 23 or 24 or 25 or 26 or 27 or 28 or 29 or 30 or 31 or 32 or 33 (19902)

35 6 and 34 (303)

**Database: Ovid MEDLINE(R) and In-Process, In-Data-Review & Other Non-Indexed Citations <1946 to** **September 13, 2021> Search Strategy**:

--------------------------------------------------------------------------------

1 "Africa south of the Sahara"/ or *South Africa/ or *Africa/ or *North Africa/ or *Central Africa/ (12091)

2 (Algeria or Angola or Benin or Botswana or "Burkina Faso" or "Burkina Fasso" or "Upper Volta" or Burundi or Urundi or Cameroon or Cameroons or Cameron or Camerons or "Cape Verde" or "Central African Republic" or Chad or Comoros or "Comoro Islands" or Comores or Congo or Zaire or "Cote d'Ivoire" or "Ivory Coast").hw,kf,ti,ab,cp. (55280)

3 (Madagascar or Malawi or Nyasaland or "Mali Marshall Islands" or Mauritania or Mauritius or "Agalega Islands" or Morocco or Mozambique or Namibia or Niger or Nigeria or "Northern Mariana Islands").hw,kf,ti,ab,cp. (93710)

4 (Rwanda or Ruanda or "Sao Tome" or Senegal or Seychelles or "Sierra Leone" or Somalia or Sudan or Suriname or Surinam or "South Sudan Swaziland" or Eswatini or Tanzania or Togo or "Togolese Republic" or Tunisia or Uganda or Zambia or Zimbabwe or rhodesia).hw,kf,ti,ab,cp. (108703)

5 (Djibouti or Egypt or Eritrea or Ethiopia or Gabon or "Gabonese Republic" or Gambia or Ghana or "Gold Coast" or Guinea or Kenya or Lesotho or Basutoland or Liberia or Libya).hw,kf,ti,ab,cp. (304397)

6 1 or 2 or 3 or 4 or 5 (534353)

7 (strategic adj8 purchas*).ti,ab,kw. (139)

8 (Quality-based adj8 purchas*).ti,ab,kw. (6)

9 (Quality adj8 based adj8 purchas*).ti,ab,kw. (115)

10 (Performance-based adj8 purchas*).ti,ab,kw. (10)

11 (Performance adj8 based adj8 purchas*).ti,ab,kw. (89)

12 (result? adj8 based adj8 purchas*).ti,ab,kw. (57)

13 (result-based adj8 financ*).ti,ab,kw. (7)

14 (result? adj8 based adj8 financ*).ti,ab,kw. (209)

15 Fee-for-service.ti,ab,kw. (5908)

16 pay-for-performance.ti,ab,kw. (2166)

17 (provider-payment adj8 reform?).ti,ab,kw. (56)

18 (provider adj8 payment adj8 reform?).ti,ab,kw. (74)

19 (Strategic adj8 contract*).ti,ab,kw. (56)

20 Bundled-Paym*.ti,ab,kw. (1044)

21 (Value-based adj8 payment?).ti,ab,kw. (715)

22 (value-based adj8 purchas*).ti,ab,kw. (530)

23 (Purchas* adj8 reform?).ti,ab,kw. (130)

24 (GP adj8 contract*).ti,ab,kw. (227)

25 (hospital adj8 contract*).ti,ab,kw. (1229)

26 (provider adj8 contract*).ti,ab,kw. (435)

27 capitation.ti,ab,kw. (2536)

28 payment-per-case.ti,ab,kw. (12)

29 (contracting adj8 mechanism).ti,ab,kw. (92)

30 (contract* adj8 health adj8 servic*).ti,ab,kw. (649)

31 (purchas* adj8 mechanism*).ti,ab,kw. (156)

32 (contract* adj8 arrangem*).ti,ab,kw. (571)

33 7 or 8 or 9 or 10 or 11 or 12 or 13 or 14 or 15 or 16 or 17 or 18 or 19 or 20 or 21 or 22 or 23 or 24 or 25 or 26 or 27 or 28 or 29 or 30 or 31 or 32 (15315)

34 6 and 33 (272)

**PubMed/Econ lit**

((((((((((("strategic purchasing"[Title/Abstract]) OR ("quality-based purchasing"[Title/Abstract])) OR ("performance-based purchasing"[Title/Abstract])) OR ("result-based financing"[Title/Abstract])) OR ("fee-for-service"[Title/Abstract])) OR ("pay-for-performance"[Title/Abstract])) OR ("provider payment reform?"[Title/Abstract])) OR ("strategic contracting"[Title/Abstract])) OR ("bundled-payment?"[Title/Abstract])) OR ("value-based payment?"[Title/Abstract])) OR (capitation[Title/Abstract])) AND (Africa or algeria or Angola or Benin or Botswana or "Burkina Faso"[Title/Abstract] or "Burkina Fasso"[Title/Abstract] or "Upper Volta"[Title/Abstract] or Burundi or Urundi or Cameroon or Cameroons or Cameron or Camerons or "Cape Verde"[Title/Abstract] or "Central African Republic"[Title/Abstract] or Chad or Comoros or "Comoro Islands"[Title/Abstract] or Comores or Congo or Zaire or "Cote d'Ivoire"[Title/Abstract] or "Ivory Coast"[Title/Abstract] or Djibouti or Egypt or Eritrea or Ethiopia or Gabon or "Gabonese Republic"[Title/Abstract] or Gambia or Ghana or "Gold Coast"[Title/Abstract] or Guinea or Kenya or Lesotho or Basutoland or Liberia or Libya or Madagascar or Malawi or Nyasaland or "Mali Marshall Islands"[Title/Abstract] or Mauritania or Mauritius or "Agalega Islands"[Title/Abstract] or Morocco or Mozambique or Namibia or Niger or Nigeria or "Northern Mariana Islands"[Title/Abstract] or Rwanda or Ruanda or "Sao Tome"[Title/Abstract] or Senegal or Seychelles or "Sierra Leone"[Title/Abstract] or Somalia or Sudan or Suriname or Surinam or "South Sudan Swaziland"[Title/Abstract] or Eswatini or Tanzania or Togo or "Togolese Republic"[Title/Abstract] or Tunisia or Uganda or Zambia or Zimbabwe or rhodesia)

**World Bank Open Knowledge repository**

result-based financ* Or Performance-based financ* OR pay-for-performance OR "strategic purchasing" Or disbursed-linked result* AND Africa

**Supplementary Appendix 2: Stage I and II categories of the studies**

**Stage I**

1. The study reports on strategic purchasing for healthcare in Africa (108),
2. The study reports on strategic purchasing for healthcare across continents including Africa (14),
3. The study may have important information on strategic purchasing for healthcare in Africa but does not belong to categories “A” or “B” (21),
4. The study was not relevant to strategic purchasing for healthcare in Africa (515),
5. The report of the study was not written in English language (6).

**Stage II**

1. Full scientific journal article on strategic purchasing for healthcare in Africa (73),
2. Multi-country scientific journal article on strategic purchasing for healthcare that included data from an African country (7),
3. Multi-country study that did not have data on strategic purchasing for healthcare from an Africa country (5),
4. Conference abstract, background article or commentary (29),
5. Not relevant to strategic purchasing for healthcare in Africa (29).

**Supplementary Appendix 3: Definitions for the 23 key roles of governments, providers, and purchasers in relation to citizens**

Source: Mbau et al (2018) and RESYST (2014).

**Key roles of governments in relation to purchasers**

In strategic purchasing, governments are expected to provide the regulatory framework and direction to facilitate the strategic purchasing arrangements (Ayako Honda, 2016). The specific roles of governments are (Role 1-4):

1. Establish frameworks: governments have to ensure that there are strategic purchasing frameworks for both purchasers and providers that are clearly stipulated in laws and policies on strategic purchasing.
2. Fill service delivery infrastructure gaps: governments are expected to ensure that there are adequate service delivery infrastructure that support strategic purchasing such as infrastructure: may include availability of health facilities that have enough health care workers, medicines and health commodities.
3. Ensure adequate resources are mobilized: governments should ensure that there are enough resources to meet the demand for healthcare services.
4. Ensure accountability of purchasers: governments should ensure that that there are accountability mechanisms for purchasers.

**Key roles of strategic purchasing of purchasers in relation to providers**

Strategic purchasing should define the basket of services to be rendered to the citizens and these services should be cost-effective and updated periodically. Although the government may lead but this is a key role by the purchasers (RESYST 2014). The purchaser should put in place effective channels to ensure that the needs of the citizens/population are reflected in the services offered by the providers. The system should also ensure efficiency in resource use and provide channels to allow the providers to be accountable to the population they serve (Ayako Honda, 2016). Having identified the services, the purchaser should put in place systematic methods for identifying and contracting providers of the services RESYST (2014). The specific roles are as follows (Role 5 -18):

1. Select providers: Purchasers are expected to select providers using systematic methods to ensure that a wide range of defined services are provided.
2. Agree contracts with providers: Once providers are selected, the purchasers must sign legally binding contracts or service agreements with the providers that define the quantity and quality of services the providers are expected to provide.
3. Develop list of medicines that providers can prescribe, medical supplies and standard treatments guidelines: purchasers are supposed to provide list of drugs that can be prescribed, list of medical supplies that will be reimbursed and treatment guidelines to be used by all contracted providers in order to ensure efficient utilization of resources. The guidelines are supposed to be regularly updated.
4. Manage provider payment methods: purchasers should design, implement and regularly modify provider payment methods that promote efficient service delivery.
5. Set provider payment rates: Purchasers should put in place provider payment rates that are appropriate and promote efficient service delivery.
6. Secure information on provider’s services: Purchasers should put in place mechanisms for securing information on the services rendered by the providers.
7. Monitor provider performance: purchasers are expected to monitor whether the providers are offering the services according to the agreed contracts and correct poor performance.
8. Audit claims: purchasers are expected to audit provider’s claims to ensure that only legit claims are reimbursed.
9. Protect against fraud: providers should put in place mechanisms for protecting resources against fraud and corruption.
10. Ensure providers are paid regularly: purchasers should ensure that providers are paid regularly as stipulated in the contracts.
11. Equitable allocation of resources: purchasers should ensure that resources are equitably allocated.
12. Other equitable considerations: purchasers are expected to implement other strategies to promote equitable allocation of resources.
13. Monitor user payment policies: purchasers should put in place mechanisms for monitoring payments made by the patients to the providers.
14. Information systems: purchasers are should manage information systems for monitoring services provided and reported by the providers.

**Key roles of purchasers in relation to population served or citizens**

Purchasers are expected to put in place mechanisms to ensure that the services offered by the providers reflect the choices of the population being served (Ayako Honda, 2016). The roles are (Role 19-23):

1. Identify healthcare needs of the population: purchasers should assess and identify the healthcare service delivery needs of the population including preferences and values and specify the healthcare package to be provided.
2. Inform the population of their packages: Purchasers should inform the population/citizens of their service entitlements and responsibilities.
3. Ensure the citizens access their packages: purchasers should ensure that the citizens are able to access the services they need.
4. Establish complaints and feedback mechanisms: purchasers should ensure that there are mechanisms for soliciting feedback and complaints from the citizens/population regarding the services they receive.
5. Financial accountability: purchasers should openly report on their use of the resources and how the fund is performing.

**Supplementary Appendix 4: countries where RBF was practiced**

Burundi, Tanzania, Zambia, Malawi, Democratic Republic of Congo, Egypt, Rwanda, Cameroon, Kenya, Nigeria, Uganda, Republic of Congo, Benin, Mozambique, Zimbabwe, Burkina Faso, Lesotho, and Gambia

Table S1: Studies included in the review

| **No** | **Author (Year)** | **Country** | **Methods** | **Data** | **Strategic purchasing arrangement** |
| --- | --- | --- | --- | --- | --- |
| 1 | Abiiro, Alatinga et al (2021) | Ghana | Qualitative data analysis | Media reports and stakeholder and FGD interviews | Accrediting private and public facilities to provide services to the NHIS |
| 2 | Aboagye (2013) | Ghana | Qualitative data analysis | Media reports and published documents | Accrediting private and public facilities to provide services to the NHIS |
| 3 | Agyei-Baffour, Oppong et al (2013) | Ghana | Qualitative analysis | In-depth interviews | Accrediting private and public facilities to provide services to the NHIS |
| 4 | Andoh-Adjei, Boudewijns et al (2018) | Ghana | Regression analysis | Hospital data and statistical records from statistical office | Accrediting private and public facilities to provide services to the NHIS |
| 5 | Andoh-Adjei, Spaan et al (2016) | Ghana and LMICs | Systematic review of literature | Published studies | Accrediting private and public facilities to provide services to the NHIS |
| 6 | Andoh-Adjei, Nsiah-Boateng et al (2018) | Ghana | Regression analysis | Survey of NHIS subscribers and healthcare providers | Accrediting private and public facilities to provide services to the NHIS |
| 7 | Andoh-Adjei, Cornelissen et al (2016) | Ghana | Descriptive data analysis | Survey of NHIS subscribers and healthcare providers | Accrediting private and public facilities to provide services to the NHIS |
| 8 | Andoh-Adjei, van der Wal et al (2018) | Ghana | Trend analysis | In-depth interviews | Accrediting private and public facilities to provide services to the NHIS |
| 9 | Andoh-Adjei, Nsiah-Boateng et al (2019) | Ghana | Descriptive data analysis and multinomial regression analysis | Interviews with providers accredited to the NHIS | Accrediting private and public facilities to provide services to the NHIS |
| 10 | Koduah, van Dijk et al (2016) | Ghana | Descriptive data analysis | In-depth interviews,  document reviews, observations and discussions | Accrediting private and public facilities to provide services to the NHIS |
| 11 | Obadha, Chuma et al (2020) | Kenya | Discrete choice experiment | Published documents, interviews with providers | Accrediting private and public facilities to provide services to the NHIF |
| 12 | Sackey and Amponsah (2017) | Ghana | Regression analysis | Published documents, interviews with providers and household heads | Accrediting private and public facilities to provide services to the NHIS |
| 13 | Volmink, Bertram et al (2014) | South Africa | Cost-effectiveness analysis | Published articles | Accrediting private and public facilities to provide services to the NHIS |
| 14 | Siita, Cox et al (2019) | Ghana | Propensity score matching | Demographic and Health Surveys | Accrediting private and public facilities to provide services to the NHIS |
| 15 | Atuoye, Vercillo et al (2016) | Ghana | Descriptive analysis | Grey and published literature | Accrediting private and public facilities to provide services to the NHIS |
| 16 | Ogbuabor and Onwujekwe (2018) | Nigeria | Qualitative study | In-depth interviews | Contracting providers to provide maternal and child healthcare |
| 17 | Hongoro, Funani et al (2015) | South Africa | Qualitative analysis | In-depth interviewers with GPs | Contracting private GPs to provide services to the public |
| 18 | Maluka, Chitama et al (2018) | Tanzania | Qualitative analysis | In-depth interviewers with key-informants and review of various documents | Contracting-out non-state facilities to provide primary healthcare |
| 19 | Mureithi, Burnett et al (2018) | South Africa | Qualitative analysis | In-depth interviewers with key informants and published documents | Contracting private GPs to provide primary care to the public |
| 20 | Rao, Paina et al (2018) | LMICs including Tanzania, South Africa, Uganda and Ghana | Qualitative analysis | Published articles and official documents | Contracting non-state providers |
| 21 | Siddiqi, Masud et al (2006) | Eastern Mediterranean Region including Tunisia, Morocco, Egypt | Qualitative analysis | Published articles and official documents and interviews with Ministries of Health and the private sector | Contracting non-state providers |
| 22 | Surender, Van Niekerk et al (2016) | South Africa | Qualitative analysis | In-depth interviews | Contracting private GPs |
| 23 | Vian, McIntosh et al (2015) | Lesotho | Descriptive analysis | In-depth interviews and observational data and hospital documents | Public–private  partnership (PPP) to build and manage hospitals |
| 24 | Etiaba, Onwujekwe et al (2018) | Nigeria | Descriptive analysis | In-depth interviews and focused group discussions | Contracting public and private facilities to provide services to the NHIS |
| 25 | Bertone and Meessen (2013) | Burundi | Qualitative analysis | Project documents and in-depth interviews of key informants. | Paying financial rewards to facilities based on agreed targets |
| 26 | Binyaruka, Robberstad et al (2018) | Tanzania | Regression analysis | Household survey | Paying financial rewards to facilities based on agreed targets |
| 27 | Binyaruka and Anselmi (2020) | Tanzania | Data envelopment analysis (DEA) | Health facility surveys | Paying financial rewards to facilities based on agreed targets |
| 28 | Borghi, Little et al (2015) | Tanzania | Regression analysis and cost-effectiveness analysis | Financial accounts, interviews and project records | Paying financial rewards to facilities based on agreed targets |
| 29 | Chansa, Makanu et al (2020) | Zambia | Trend analysis and qualitative analysis | Policy documents, district expenditure records, and demographic and health surveys | Contracting public facilities to provide services purchased by the MoH |
| 30 | Chimhutu Lindkvist et al (2014) | Tanzania | Qualitative analysis | In-depth interviews with health workers | Paying financial rewards to facilities based on agreed targets |
| 31 | Chimhutu, Songstad et al (2016) | Tanzania | Qualitative analysis | In-depth interviews with health workers and focus group discussions | Paying financial rewards to facilities based on agreed targets |
| 32 | Chimhutu, Tjomsland et al (2019) | Tanzania | Qualitative analysis | In-depth interviews and focus group discussions | Paying financial rewards to facilities based on agreed targets |
| 33 | Chinkhumba, De Allegri et al (2017) | Malawi | Regression analysis | Household surveys | Paying financial rewards to facilities based on agreed targets and providing financial incentives to women for deliver at health facilities |
| 34 | Chinkhumba, De Allegri et al (2020) | Malawi | Cost-effectiveness analysis | Published literature, RBF programme costs | Paying financial rewards to facilities based on agreed targets and providing financial incentives to women for deliver at health facilities |
| 35 | Das, Gopalan et al (2016) | LMICs including Burundi, Democratic Republic of Congo, Egypt and Rwanda | Systematic review of literature | Published studies | Giving bonuses to healthcare providers in return for agreed maternal, child health volume and quality services |
| 36 | Honda (2012) | LMICs including Rwanda | Review of literature | Published studies | Paying financial rewards to facilities based on agreed targets |
| 37 | Meessen, Musango et al (2006) | Rwanda | Descriptive study | Health facility data | Paying financial rewards to facilities based on agreed targets |
| 38 | Basinga Gertler et al (2011) | Rwanda | Regression analysis | Health facility and household surveys | Paying financial rewards to facilities based on agreed targets |
| 39 | Manga, Fouda et al (2018) | Cameroon | Descriptive analysis | Health worker surveys | Paying financial and non-financial bonuses to facilities and health workers based on agreed targets |
| 40 | Manongi Mushi et al (2014) | Tanzania | Qualitative study | In-depth interviews with key informants and FGD | Paying financial rewards to government and faith-based facilities based on agreed targets |
| 41 | Menya, Platt et al (2015) | Kenya | Cluster randomisation study | Hospital records | Paying financial rewards to government primary healthcare facilities based on agreed targets |
| 42 | Ngo, Sherry et al (2017) | Rwanda | Regression analysis | Service Provision Assessment  (SPA) Survey and DHS | Paying financial rewards to government facilities based on agreed quantity and quality targets |
| 43 | Ogundeji, Jackson et al (2016) | Nigeria | Qualitative study | Interviews with health workers | Paying financial rewards to facilities based on agreed targets |
| 44 | Witter Fretheim et al (2012) | LMICs including Rwanda, Tanzania, Uganda, Democratic Republic of Congo and Burundi | Cochrane systematic review | Published studies | Paying financial rewards to facilities based on agreed targets |
| 45 | De Allegri, Chase et al (2019) | Malawi | Regression analysis | Health facility data | Paying financial rewards to public facilities based on agreed targets and cash transfers to service users |
| 46 | De Allegri, Makwero et al (2019) | Malawi | Costing study | Programme documents | Paying financial rewards to public facilities based on agreed targets and cash transfers to service users |
| 47 | Fritsche and Peabody (2018) | LMICs including Democratic Republic of Congo and Republic of Congo | Qualitative study | PBF project data | Contracting public and private facilities in RBF programme |
| 48 | James, Lawson et al (2020) | LMICs including Benin,  Burundi, Cameroon, Democratic Republic of Congo,  Mozambique, Zambia, Zimbabwe and Nigeria | Systematic review of literature | Published and grey literature | RBF for Maternal Neonatal and Child Health services |
| 50 | Kane, Gandidzanwa et al (2019) | Zimbabwe | Qualitative study | In-depth interviews with health workers | Paying financial rewards to facilities based on agreed targets |
| 51 | Lohmann, Wilhelm et al (2018) | Malawi | Qualitative study | In-depth interviews with health workers | Paying financial rewards to public facilities based on agreed targets and cash transfers to service users |
| 52 | Paul, Brown et al (2020) | Zambia | A critique | Published paper | Paying financial rewards to public facilities based on agreed targets |
| 53 | Rajkotia, Zang et al (2017) | Mozambique | Regression analysis | PBF invoices, health facility and national surveys data | Paying financial rewards to public facilities based on agreed targets |
| 54 | Ridde, Gautier et al (2018) | Africa including Benin, Burkina Faso,  Cameroon, Lesotho, and Mozambique | Review article | Published studies | Paying financial rewards to facilities based on agreed targets |
| 55 | Seppey, Ridde et al (2020) | Burkina Faso | Qualitative study | In-depth interviews with health workers | Paying financial rewards to facilities based on agreed targets including subsidising services for the poor and equity bonuses for facilities working with the poor |
| 56 | Shen, Nguyen et al (2017) | Zambia | Regression analysis and qualitative analysis | Health facility and health worker surveys | Paying rewards to public facilities and health workers based on agreed quantity and quality targets |
| 57 | Sieleunou, Turcotte-Tremblay et al (2017) | Cameroon | Qualitative analysis | In-depth interviews and project documents | Paying rewards to facilities on agreed targets |
| 58 | Skiles, Curtis et al (2015) | Rwanda | Regression analysis | Demographic and health surveys | Paying rewards to public facilities and health workers based on agreed quantity and quality targets |
| 59 | Soeters, Habineza et al (2006) | Rwanda | Qualitative analysis | Household surveys | Paying rewards to facilities and health workers based on agreed quantity and quality targets |
| 60 | Ssengooba, Ssennyonjo et al (2021) | Uganda | Qualitative analysis | In-depth interviews, grey and published literature | Paying rewards to public and private facilities and health workers based on agreed targets |
| 61 | Ssennyonjo, Ekirapa–Kiracho et al (2021) | Uganda | Qualitative analysis | In-depth interviews, grey and published literature | Paying rewards to public and private facilities and health workers based on agreed targets |
| 62 | Wilhelm, Brenner et al (2016) | Malawi | Qualitative analysis | In-depth interviews with key informants | Paying financial rewards to public facilities based on agreed targets and cash transfers to service users |
| 63 | Witter, Bertone et al (2019) | Zimbabwe, Uganda, DRC | Qualitative analysis | In-depth interviews, grey and published literature | Paying financial rewards to public and non-governmental facilities based on agreed targets |
| 64 | Witter, Chirwa et al (2019) | Zimbabwe | Qualitative analysis | In-depth interviews and project documents | Paying financial rewards to public and non-governmental facilities based on agreed targets |
| 65 | Witter, Chirwa et al (2020) | Zimbabwe | Qualitative analysis | In-depth interviews, grey and published literature | Paying financial rewards to providers based on agreed targets |
| 66 | Zeng, Shepard et al (2018) | Zambia | Cost-effectiveness analysis | National  pharmaceutical distribution hub costs, RBF programme costs, household surveys | Paying financial rewards to providers based on agreed targets |
| 67 | Zizien, Korachais et al (2019) | Burkina Faso | Regression analysis | Statistical yearbooks and the national health data warehouse | Paying financial rewards to providers based on agreed quantity and quality targets |
| 68 | Fichera, Anselmi et al (2021) | Zimbabwe | Regression analysis | Demographic and Health Survey (DHS) | RBF in health facilities were paid based on agreed indicators |
| 69 | Sieleunou, Tamga et al (2021) | Cameroon | Scooping literature review | Published articles including grey literature | All strategic purchasing arrangements |
| 70 | Binagwaho Condo et al (2014) | Rwanda | Regression analysis | General Health and  HIV household survey | Paying financial rewards to providers based on agreed targets |
| 71 | Mabuchi, Sesan et al (2018) | Nigeria | Qualitative study | In-depth interviews, FGD, health facility and observational data | Paying financial rewards to providers based on agreed quantity and quality targets |
| 72 | Steenland, Robyn et al (2017) | Burkina Faso | Regression analysis | Health facility data | Contracting public facilities to receive financial rewards to providers based on agreed quantity and quality targets |
| 73 | Antony, Bertone et al (2017) | Benin | Qualitative study | Published documents, project documents, FGDs, key informant interviews | Paying financial rewards to public facilities based on agreed targets |
| 74 | Agyepong, Aryeetey et al (2014) | Ghana | Trend and descriptive analysis | Published studies, hospital data and in-depth interviews with key informants | Accrediting private and public facilities to provide services to the NHIS |
| 75 | Mohammed, Souares et al (2014) | Nigeria | Qualitative study | Interviews with providers | Accrediting private and public facilities to provide services to the NHIS |
| 76 | Obadha, Chuma et al (2019) | Kenya | Qualitative study | Interviews with providers | Accrediting private and public facilities to provide services to the NHIF or private insurers |
| 77 | Robyn, Bärnighausen et al (2012) | Burkina Faso | Discrete choice experiment | Interviews with providers | Contracting using community health insurance funds paid capitation |
| 78 | Robyn, Bärnighausen et al (2014) | Burkina Faso | Qualitative and quantitative data analysis | Cross-sectional interviews | Contracting using community health insurance funds paid capitation |
| 79 | De Walque, Gertler et al (2015) | Rwanda | Regression analysis | Health facility surveys | Paying financial rewards to government facilities based on agreed quantity and quality targets |
| 80 | Ferguson, Hasan et al (2020) | Gambia | Qualitative analysis | Primary data collected from communities | RBF in health facilities for Maternal and Child Nutrition and Health |
